# Taming Mre11–Rad50 at Telomeres: Convergent Strategies for Chromosome‐End Protection Across Evolution

**DOI:** 10.1002/bies.70197

**Published:** 2026-10-21

**Authors:** Florian Roisné‐Hamelin, Stephan Gruber

**Affiliations:** ^1^ Department of Fundamental Microbiology (DMF) Faculty of Biology and Medicine (FBM) University of Lausanne Lausanne Switzerland

**Keywords:** end protection problem, end replication problem, MRN, MRX, Phage N15, Rif2, SbcCD, TelN, telomerase, telomere

## Abstract

Telomere biology is often framed as a uniquely eukaryotic problem, yet the challenges of maintaining DNA ends are shared by all organisms harboring linear DNA, including viral entities. Telomeres enable complete replication of chromosome ends while shielding the termini from DNA repair factors and innate immune surveillance systems that would otherwise recognize them as damaged or foreign DNA. A particular threat is posed by Mre11‐Rad50, a nuclease complex conserved from bacteria to humans that specializes in the detection and processing of DNA ends. The solutions to these DNA end‐related challenges that have emerged across the tree of life converge on a small number of recurring strategies. Here, we review how diverse biological systems, from bacteriophages to mammals, have solved the DNA end‐related challenges, with a particular focus on recent advances in our understanding of end protection.

## Graphical abstract

All telomeres must be protected from DNA repair enzymes. This review explores how, from bacteriophages to mammals, unrelated telomere proteins have converged on the same recurring strategies: inhibiting the Mre11–Rad50 nuclease directly or physically shielding DNA ends.

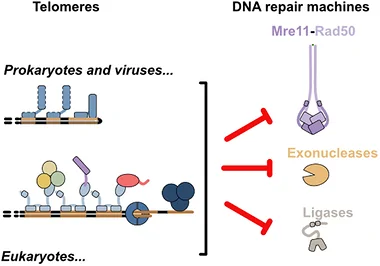

## 1 Introduction

All linear chromosomes require specialized nucleoprotein structures at the DNA ends called telomeres which fulfill two functions critical for maintaining the integrity of chromosome extremities. First, they provide a solution to the so‐called “end replication problem”, the inability of semiconservative replication to fully replicate the 3’ end of DNA strands, either through direct structural mechanisms that prevent shortening altogether (in some prokaryotes and viruses), or, in most eukaryotes, through the recruitment and regulation of a dedicated reverse transcriptase, telomerase, that counteracts shortening. Second, they solve the “end protection problem” by shielding the termini of linear chromosomes from the DNA repair machinery, which specializes in recognizing and processing (e.g., resecting or ligating) DNA ends. Failure of this essential function has drastic consequences for chromosome integrity, including chromosome fusions and gross chromosomal rearrangements [1, 2, 3, 4, 5].

While bacterial and archaeal genomes are typically—though not always—circular, eukaryotes organize their genetic information into linear chromosomes. What explains these opposing strategies for genome organization? Despite the inherent structural challenges of telomere end‐maintenance, eukaryotes generally avoid circular chromosomes. During homologous recombination, circular DNA molecules readily fuse into dimeric rings. Bacteria utilize dedicated, site‐specific recombination systems to resolve these dimers, however, such systems are fundamentally incompatible with eukaryotic meiosis, where stable crossovers are required for proper chromosome segregation [6, 7]. Genome engineering in budding yeast highlights this constraint. Strains with a circular chromosome often fail to execute meiosis likely due to the formation of dimeric (and thus dicentric) chromosomes [8, 9, 10]. This suggests that circularity of a single chromosome can disrupt eukaryotic sexual reproduction. Thus, while the linear nature of eukaryotic chromosomes creates end‐maintenance vulnerabilities, it confers an enormous evolutionary advantage: compatibility with the complex crossover events of meiosis, which drives genetic diversity and adaptability. Consistent with this line of thought, the linear ends of certain bacterial chromosomes, such as those in the *Borrelia* genus, have also been associated with elevated genetic recombination and rapid antigenic variation to evade host immunity [11]. Most bacteria, however, maintain circular chromosomes [12]. In addition to avoiding end‐related challenges, the absence of telomeres may enhance the ability of their innate immune systems to recognize mobile genetic elements (MGE) with linear genomes. Indeed, DNA end sensing is a prominent feature in bacterial defense systems and also plays a role in viral restriction (i.e., the cellular process by which host proteins block or limit viral infection and replication). The evolutionary origins of telomere maintenance itself appear deeply entangled with immune defense (discussed below). Bacteriophage genomes and plasmids can also be linear. Although archaea are thought to lack telomeres, viruses of archaea can harbor linear DNA molecules. These mobile genetic elements have evolved dedicated telomeric structures at their genome termini, essential for genome maintenance [13]. Even eukaryotic viruses, such as adenoviruses, have evolved specialized mechanisms to protect their DNA ends [3, 14, 15]. Together, these systems point to a fundamental and ancient biological challenge that has been solved independently multiple times across the tree of life with the solutions showing recurring themes.

Telomere protection relies on multiple complementary pathways acting synergistically, ensuring both robust and adaptable protection across the phases of the cell cycle. Telomeres must neutralize multiple components of the DNA repair machinery, including homologous recombination (HR) and non‐homologous end joining (NHEJ) pathways and DNA‐damage signaling factors, as well as the innate immune system [1, 2, 3, 4, 5]. Paradoxically, telomeres also co‐opt certain repair proteins for their own physiology, for example, to prevent nucleolytic processing by exonucleases. Evolution has produced distinct peptide motifs on unrelated telomere proteins that all converge on direct inhibition of the MR (Mre11‐Rad50) nuclease complex, a central regulator of DNA repair and DNA end sensing [16]. Recent work on bacteriophage genomes has further identified bacterial MR as a target of a dedicated phage‐encoded protein [17], indicating that convergent strategies for neutralizing this complex have arisen across domains of life, possibly reflecting a universal constraint of end protection.

Here, we review how telomere structure and associated mechanisms have evolved across the tree of life, with a focus on the shared strategies and distinct molecular solutions that different lineages have deployed to solve end‐related problems. We further discuss how telomeres function as molecular signals at the interface of genome integrity and innate immune surveillance, and how biological conflicts—between host and pathogen, between selfish genetic elements and the genome—may have driven the evolution of telomere protection strategies. We first briefly introduce various solutions for end replication and then mainly focus on end protection. As this review centers on comparing telomere protection mechanisms across evolution, we will refer interested readers to specialized reviews for organism‐ or mechanism‐specific detail beyond our comparative scope, wherever appropriate.

## 2 How Telomere Sequence and Structure Support Replication at Chromosome Ends

The end replication problem is the inability to fully replicate chromosome ends [18], resulting in progressive end shortening without dedicated countermeasures. It arises from the requirement for an RNA primer during DNA synthesis of the lagging strand, resulting in incomplete replication at the 3' end upon primer removal. This problem is potentially inherent to any linear DNA molecule replicated by conventional RNA‐primed DNA synthesis [19], and thus applies broadly across bacteria, archaea, eukaryotes, and their viruses. Two strategies have evolved in response to this challenge. One prevents shortening altogether, through DNA hairpin ends or protein‐based priming [12, 20, 21], the other instead counteracts telomere shortening, through recombination‐based sequence addition or RNA‐templated extension of the 3' end using a dedicated enzyme, the telomerase [22, 23, 24, 25].

In most eukaryotes, the overall organization of telomere architecture is conserved. Telomeres are typically formed by tandem repeat DNA sequences recognized by a first layer of Myb‐domain proteins (TRF1/2 in mammals, Rap1 in budding yeast), which recruits a dynamic set of telomere proteins.

In budding yeast *Saccharomyces cerevisiae*—a pioneer organism in telomere research—telomeres consist of approximately 300 bp (± 75) of irregular TG_1‐3_ repeats, terminated by a short 3' ssDNA overhang of 0–15 nucleotides (nt) [4, 5]. Mammalian telomeres follow the same typical organization but at a greater scale and complexity. Human telomeres span approximately 15 kb of more regular T_2_AG_3_ repeats, terminated by a 3' ssDNA overhang estimated to 50–400 nt, while laboratory mouse telomeres can reach up to 100 kb [1, 26, 27]. Meanwhile, fission yeast (*Schizosaccharomyces pombe*) telomeres maintain a length of ∼300 bp [28].

Telomerase counteracts telomere shortening in most eukaryotes (Figure 1A), although the enzyme has been lost in rare cases [29, 30], for example in Diptera (see below). This specialized reverse transcriptase uses an RNA template contained in a long non‐coding RNA (TERC for Telomerase RNA component; 451 nt in humans, of which only 11 nt serve as template) to synthesize DNA and elongate the 3′ end; the standard replication machinery then extends the complementary 5′ strand [22, 23, 24, 31, 32]. The level of detectable telomerase activity varies across species, cell types, and tissues. Although yeast (or mice) telomerase is generally active, albeit tightly regulated (by telomere proteins), in humans, telomerase activity is restricted to the germline, stem cells and activated lymphocytes, while most somatic cells lack telomerase activity [4, 5, 33]. The lack of telomerase activity results in progressive telomere shortening due to the above‐described end replication problem. This erosion ultimately compromises telomere protection (discussed below), triggering a checkpoint response that permanently arrests the cell cycle before chromosome integrity becomes compromised. This process is called replicative senescence and has been detected in both yeast (telomerase‐null) [34] and mammalian cells [35, 36] where it contributes to tissue aging [37]. Senescence can eventually be bypassed by a recombination‐based alternative lengthening of telomeres (ALT) pathway (first detected in yeast [38] then in mammals [39]), which, together with telomerase reactivation can restore cell proliferation capacity in human cancer cells [1, 40]. *Drosophila melanogaster* represents a striking exception to canonical eukaryotic telomere organization. Rather than telomerase‐synthesized simple repeats, *D. melanogaster* chromosome ends are maintained by the retrotransposition of three non‐LTR retrotransposons—HeT‐A, TART, and TAHRE—onto chromosome termini [41, 42, 43].

**FIGURE 1:**
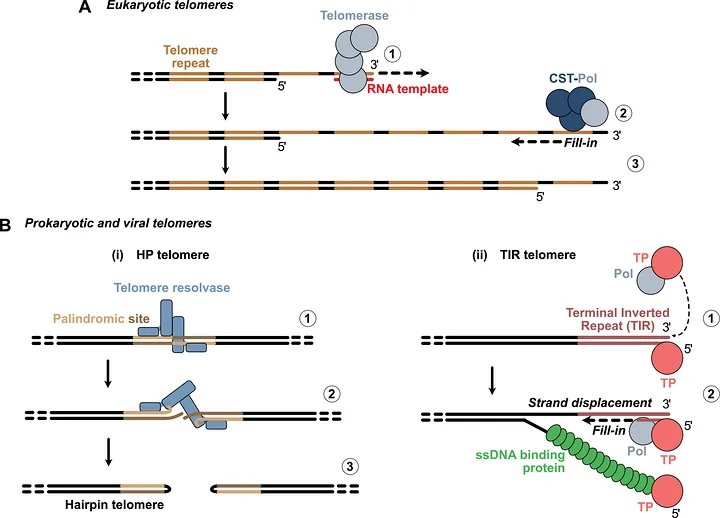
Telomere replication strategies across evolution. (A) In most eukaryotes, the telomerase adds new telomere sequence repeats to the 3' strand using its reverse transcriptase activity (1). The CST (CTC1‐STN1‐TEN1 in humans) complex binds to single‐stranded telomeric DNA and recruits the DNA polymerase α‐primase to synthetize the complementary strand [1, 4, 24]. (B) Two types of bacterial and viral telomere replication strategies have been described: hairpin (HP) and terminal‐protein based (TIR). (i) For hairpin telomeres (HP), the telomere resolvase recognizes the palindromic site on a circular genome intermediate generated by conventional replication from an internal origin (1) and performs the cleaving joining reaction (2) to produce two new hairpin telomeres (3) [21]. The palindromic telomere sequence *tos* (*Telomerase Occupancy Site*) is required to support telomere resolution [44]. The palindromic site is created again by the subsequent round of DNA replication. (1) (ii) For terminal‐protein‐based terminal‐inverted‐repeat (TIR) telomeres, the terminal protein recruits a DNA polymerase and prime DNA replication (1) of the whole genome by strand displacement (2) [45]. For simplicity, the second variant of TP telomeres is not shown here [20].

In bacteria, two types of telomeres have been identified: hairpin (HP) telomeres and terminal inverted repeat (TIR, also called ITRs for inverted terminal repeats) telomeres (Figure 1B). These telomeres are also found in some bacteriophages, and some viruses infecting eukaryotes harbor TIR telomeres too. HP telomeres physically seal the DNA end by connecting the 5’ to the 3’ strand via a short loop [21]. Such telomeres are present in *Borrelia* and *Agrobacterium* (biovar I), several bacteriophages including *Klebsiella oxytoca* phage phiKO2 and the *Escherichia coli* phage N15, as well as in poxviruses [46, 47, 48, 49, 50]. Maintenance of HP telomeres depends on a dedicated enzyme, the telomere resolvase (also termed protelomerase) which recognizes specific DNA sequences found at the hairpin end. The best characterized examples are the TelN protein in coliphage N15 and ResT in *Borrelia burgdorferi* [21, 44, 51, 52]. At the telomere, DNA replication, initiated at an internal origin, converts the hairpin into a palindromic dimer made of two inverted copies of the telomere sequence. The telomere resolvase then resolves these dimeric telomere sequences through a concerted strand cleavage and rejoining reaction, termed telomere resolution, generating two hairpin ends (Figure 1B(i)) [21]. Prior to telomere resolution, the chromosome therefore temporarily exists as Y‐shaped or circular intermediate during DNA replication.

The second category of prokaryotic and viral telomeres is defined by terminal inverted repeats (TIR), sequences ranging from hundreds to thousands of base pairs with short palindromic elements, with the 5’ end being covalently bound by a dedicated terminal protein [20, 45]. Such telomeres are found on linear chromosomes and plasmids such as pSLA2 in *Streptomyces* [53, 54], in the *Bacillus* bacteriophage Phi29 [46], in the enterobacteria phage PRD1 [55], and in human adenoviruses [14, 45, 56, 57]. Two distinct solutions for the end replication problem have been described for TIR telomeres. In phage Phi29 and human adenoviruses, the terminal protein recruits a DNA polymerase and acts as a protein primer to initiate DNA synthesis of the entire genome (Figure 1B(ii)). In this process, the terminal protein (TP) become covalently bound to the newly synthesized 5’ end [58]. In *Streptomyces*, bidirectional replication from an internal origin instead initially generates 250–300 nt single‐stranded 3’ overhangs at each telomere end [53, 59]. These overhangs contain palindromic sequences capable of forming fold‐back hairpin structures that recruit TAP (a telomere‐associated protein) associated with the terminal protein (named Tpg in *Streptomyces*, after the corresponding gene) to the site of DNA synthesis [54, 60, 61, 62]. Together with DNA polymerases, these proteins then fill in the complementary strand using the 3' overhang as a template while also covalently attaching the terminal protein to the 5' end, a process termed “end patching” [20, 53, 54, 63, 64].

In summary, solving the end replication problem requires specialized machinery, typically a compact toolkit—a single protein or protein complex. In contrast, faithful end protection often demands a vast molecular network, relying on a multitude of factors and overlapping, redundant mechanisms which we describe below.

## 3 The DNA Repair Machinery Threatens Telomere Integrity

The end protection problem is, at its core, a problem of molecular recognition. The DNA repair machinery must recognize various types of DNA ends generated by DNA damage or abortive DNA transactions and must distinguish them from legitimate chromosome ends. Cellular innate immune surveillance systems must discriminate DNA signatures of foreign genetic elements [3, 65] from damaged DNA and from telomeric DNA. Telomeres help solve this problem by establishing a molecular “self and safe” signal that suppresses inappropriate repair and immune activation. Inappropriate processing of telomeres, for instance if they become critically short and therefore less effective as protective structures, can lead to catastrophic chromosomal instability [1, 2]. Viral entities harboring linear genomes face the same structural challenge but must achieve another goal: subverting both host immune defenses and DNA repair to ensure their survival and proliferation within the cell.

DNA repair nucleases are a threat to telomere integrity. Exonucleases typically resect one of the two DNA strands, starting from an exposed DNA end with the 5’ end being the prime target of resection pathways [66]. Ligases working in nonhomologous end joining [67] may lead to telomere–telomere fusion when active at telomeres. Sterically blocking access to the very end of DNA is sufficient to protect DNA ends from these enzymes (see below). A more challenging threat to telomeres, however, is posed by the Mre11‐Rad50 MR complex—SbcCD in bacteria (here denoted as MR_bact_), MRX (Mre11‐Rad50‐Xrs2) in yeast and MRN (Mre11‐Rad50‐Nbs1) in most other eukaryotes. MR is a master regulator of DSB (double‐strand break) repair specialized in the sensing, signaling and initial processing of DNA ends [16, 68, 69]. The Mre11 subunit is a multipurpose nuclease whose activity is controlled by the ATP‐binding SMC‐like (Structural Maintenance of Chromosome) coiled coil protein Rad50. Besides being a 3’‐to‐5’ exonuclease, it has endonuclease activity that is capable of cleaving DNA internally, but only in the vicinity of a DNA end. MR loads onto DNA ends using the long Rad50 coiled coils and in doing so has the remarkable ability to bypass protein obstacles at the DNA end, allowing it to endonucleolytically process various DNA ends, including those blocked by protein complexes or DNA secondary structures (Figure 2) [16, 70, 71]. Although this capability is critical for chromosome maintenance, for example, to remove NHEJ factors or to process meiotic DSBs covalently bound to Spo11 [16], this MR activity must be tightly controlled at telomeres to ensure their stability. Consistent with this view, artificial linearization of the bacterial chromosome is tolerated only when the MR_bact_ complex is inactivated or when an MR_bact_ inhibitor protects the telomere [17, 72]. End protection is therefore a critical function of any telomere (Figure 3), and likely a prerequisite for the stable coexistence of linear chromosomes and DNA repair capabilities in a cell. Blocking MR poses a significant challenge due to MR's ability to overcome protein obstacles of significant sizes. The protection strategies described below fall into two broad categories: direct inhibition of MR by protein‐protein contacts and shielding mechanisms based on steric hindrance.

**FIGURE 2:**
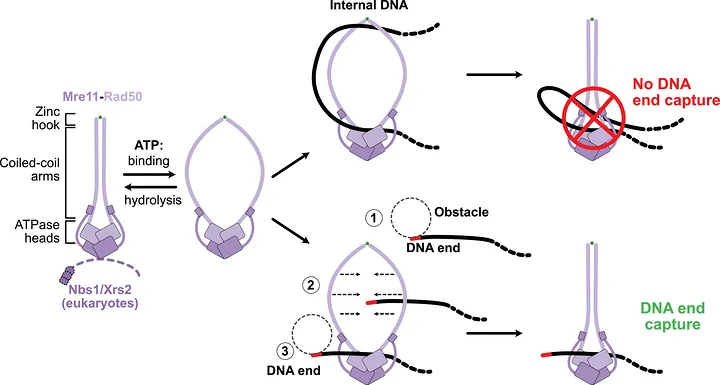
DNA end capture and protein obstacle bypass by the Mre11‐Rad50 nuclease. The MR (or MRN) complex, with Nbs1/Xrs2 being eukaryote‐specific, recognizes broken ends and coordinates their repair. In eukaryotes, MRN directly initiates homologous recombination through its nuclease activity, supports non‐homologous end joining, and activates the checkpoint kinase ATM to propagate the DNA damage signal [16, 68, 69]. MRN also contributes to antiviral defense, particularly against viruses with linear genomes [3]. In bacteria, the roles of the SbcCD complex are more restricted, primarily encompassing homologous recombination and bacteriophage elimination. MRN is structurally related to Structural Maintenance of Chromosomes (SMC) complexes, which share a characteristic ring‐shaped architecture organized around an SMC protein dimer (here, Rad50). Each SMC protomer consists of an ATPase head domain connected via a long coiled‐coil arm (spanning tens of nanometers) to a dimerization domain at the apex, which is a zinc hook in the case of Rad50 [73]. Rad50 associates with an Mre11 nuclease dimer and the eukaryote specific regulatory subunit Nbs1^Xrs2^. MRN is proposed to sense DNA ends through a conformational mechanism involving its coiled‐coil arms. ATP binding by Rad50 opens the coiled‐coil arms, enabling DNA sensing. When the complex encounters a DNA duplex internally (top), a DNA loop threaded between the coiled coils sterically prevents their closure, precluding stable loading. At a DNA end (bottom), however, only a single DNA duplex crosses between the coiled coils, permitting their closure and stable end capture [16, 74]. The aperture of the Rad50 coiled coils allows the MR complex to bypass obstacles present at DNA ends, such as other DNA repair proteins. This unique property is a threat to nonprotected telomeres.

**FIGURE 3:**
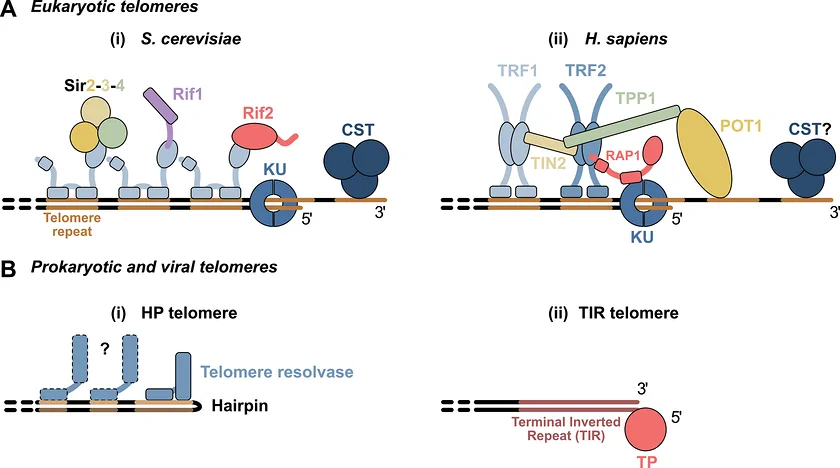
Telomere proteins across evolution. (A) Examples of eukaryotic telomere architecture. (i) In budding yeast, the telomere repeats are recognized by the Myb‐domain protein Rap1. Rap1 recruits Rif1, Rif2, Sir2, Sir3, and Sir4, generating a dynamic “molecular velcro” architecture [75]. The 3' single‐stranded overhang is bound by the RPA‐like CST complex (Cdc13‐Stn1‐Ten1) and the NHEJ factor KU (Ku70/Ku80) is recruited to yeast telomeres through two independent pathways: direct binding to the telomere tip, or interaction with Sir4 (not shown) [4, 5]. (ii) In mammals (here *H. sapiens*), telomeres are recognized by two paralogous Myb‐domain proteins, TRF1 and TRF2, which together recruit the remaining telomere proteins: RAP1, TIN2, TPP1, and POT1 (note that rodents have two paralogs of this protein, POT1a and POT1b), the latter contacting the single‐stranded overhang. KU is also recruited to telomeres, presumably at the extremity as in yeast [1]. Fission yeast (not shown) occupies an informative evolutionary position between budding yeast and mammals: a single Myb‐domain protein, Taz1 (ortholog of TRF1/2), binds telomeric repeats and recruits Rap1, Poz1, Tpz1, Pot1, and Ccq1 [28]. The molecular organization of Drosophila telomeres (not shown) remains elusive. HOAP and HipHop are thought to bind and spread across chromosome ends, where they are thought to form the terminin complex together with HP1, Ver, and Moi [76]. (B) Examples of prokaryotic and viral telomere architecture [21, 45]. (i) The telomere resolvase could possibly remain bound to the Hairpin telomere after replication. Additional copies of the telomere resolvase may bind to degenerate telomere sequence elements located in the flanking DNA. (ii) In TIR telomeres, the terminal inverted repeats are covalently linked to a protein at the 5' end of the DNA.

## 4 Protein–Protein Interactions Specifically Inhibit Mre11‐Rad50 (MR) at Eukaryotic Telomeres

Eukaryotic telomeres (Figure 3) have evolved multiple strategies to suppress DNA repair and ensure their stability. These strategies include direct inhibition of MR by protein–protein contacts and shielding mechanisms based on steric hindrance (Figure 4). The former involves a collection of dedicated peptide motifs that locally suppress MRN activity through protein–protein interactions. Such motifs have been identified in *S. cerevisiae* Rif2 (termed BAT, for Block Addition of Telomeres, or MIN, for MRX‐Inhibitory motif) [77, 78, 79, 80, 81], in *S. pombe* Taz1 [79], and in mammalian TRF2, where the equivalent motif is termed iDDR (Inhibitor of DNA Damage Response) [82, 83]. These motifs were initially identified through mutagenesis studies aimed at deciphering the mechanistic functions of TRF2 and Rif2, and they were found to be required for checkpoint suppression at telomeres [78, 83], which is mediated by ATM activation by MRN at short telomeres [1, 16]. They reside in intrinsically disordered protein regions and are predicted, or have been shown, to adopt a defined conformation upon contacting an exposed β‐sheet on the Rad50 ATPase head (Figure 4A), resulting in inhibition of most, if not all, MRN functions at telomeres [78, 79, 80, 81, 82, 83, 84, 85, 86].

**FIGURE 4:**
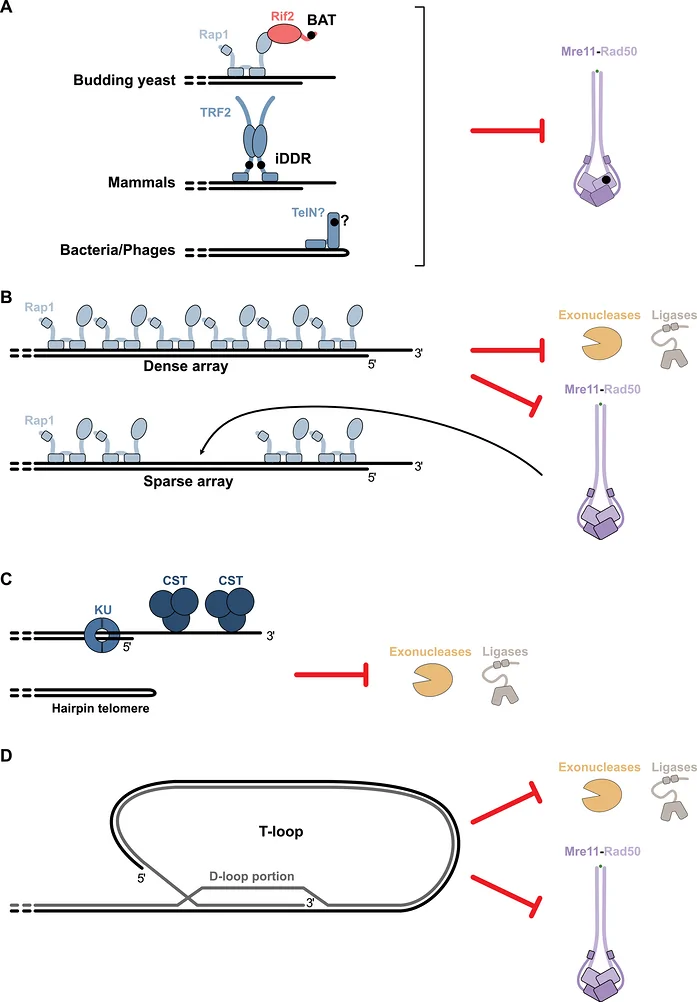
Telomere protection strategies. (A) MRN inhibition by telomere peptide motifs. BAT in Rif2 (budding yeast; MEHVDSDFAPIRRSK), MIN in Taz1 (fission yeast; DDLSEDFPPVRESD), and iDDR in TRF2 (mammals; NGVEEKETWVEEDE) are unrelated short peptide motifs that inhibit MRN at telomeres through direct interaction with Rad50. They are predicted—or shown—to bind the same region on an exposed β‐sheet of the Rad50 ATPase head (black dot). How these motifs block MRN remains to be fully deciphered; one possibility is direct competition with CtIP^Sae2^, the nuclease activator of MRN, which binds the same region of Rad50 [87]. Whether prokaryotic telomere proteins such as TelN include equivalent motifs is to be determined. (B) Myb‐domain protein coverage (here, budding yeast Rap1) protects telomeres from nucleases, including the MR complex. Efficient protection requires the Rap1 array to be dense and close to the chromosome end (top); sparse arrays instead create entry points for MR and exonuclease processing, weakening protection (bottom) [88]. Whether prokaryotic telomere proteins spread on DNA to form analogous structures is to be determined. (C) Protection of the telomere tip—for example by yeast KU/CST (top), or by prokaryotic HP (bottom) and TP telomeres (not shown)—physically blocks exonuclease (and ligases) access. (D) Higher‐order structures such as the mammalian T‐loop (whose exact architecture remains to be determined) can further restrict access of all DNA‐processing enzymes [89, 90]. The combination of multiple strategies ensures highly robust telomere protection.

These motifs target the same Rad50 surface as CtIP^Sae2/Ctp1^, the MRN resection activator thought to stabilize the cleavage‐competent state of the complex in eukaryotes [16, 66]. Accordingly, RAD50 separation‐of‐function mutants previously shown to be deficient for MRN–CtIP^Sae2/Ctp1^ nuclease activity are also refractory to telomeric inhibition [79, 80, 81, 82, 85, 86]. The BAT/MIN motif can outcompete Sae2/Ctp1 binding to Rad50, thereby suppressing Mre11 endonuclease activity. The iDDR has similarly been proposed to act by blocking CtIP access to Rad50, effectively preventing downstream MRN‐dependent resection [82, 85]. However, in budding yeast, the BAT/MIN motif also blocks MRN functions that are independent of Sae2, including NHEJ and ATM activation, and prevents stable DNA binding by MRN in vivo [77, 81]. The precise molecular mechanism by which these motifs repress MRN therefore remains to be fully elucidated.

Notably, the BAT/MIN and iDDR motifs are recruited to telomeres by unrelated proteins, share no obvious peptide sequence similarity, and are predicted to engage Rad50 in distinct orientations [82, 84, 85]. They are likewise unrelated in sequence to the CtIP^Sae2/Ctp1^ motif that contacts Rad50. The only apparent shared feature across all these motifs is an essential aromatic residue that docks onto the Rad50 head surface — a phenylalanine in BAT/MIN and CtIP/Ctp1/Sae2, and a tryptophan in iDDR. This pattern strongly suggests that MRN‐inhibitory motifs have arisen independently on multiple occasions during evolution, making telomeric MRN control a striking example of convergent molecular evolution [82]. This convergence underscores the fundamental importance of restraining MRN at chromosome ends, and it may represent one of the earliest protective functions to have emerged on linear chromosomes.

Beyond these Rad50‐directed motifs, other protein adaptors have been implicated in MRE11 regulation. GRB2 (Growth Factor Receptor‐Bound Protein 2, an adaptor protein previously known for signal transduction in the cytoplasm) has been found to promote MRE11 recruitment to double‐strand breaks through direct interactions with both MRE11 and γH2AX [91], possibly independently of the canonical MRN complex. Whether and how GRB2 might contribute to telomere regulation, and whether it acts orthogonally to the Rad50‐directed motifs described above, remains to be elucidated.

Interestingly, MRN also targets the telomeres of viral genomes, such as in adenoviruses, where it is thought to activate a local signaling response and to promote the formation of large viral genome concatemers generated by NHEJ‐mediated fusion of adenotelomeres, thereby preventing their encapsidation into virions [92, 93, 94]. Adenoviruses counteract this by deploying dedicated proteins that promote the mislocalization (E4orf3) and the degradation (E4orf6‐E1b55) of MRN subunits [93, 94, 95, 96, 97, 98, 99, 100, 101, 102, 103]— for reviews see [3, 15, 104]. Thus, these adenoviral telomere protection strategies based on protein‐induced “destruction/sequestration” of MRN fundamentally differ with the “local taming” occurring at cellular telomeres. Such destructive strategies might be tenable, possibly because viruses might have a lesser need to preserve MRN's other roles in genome maintenance. It would be intriguing to determine whether any of these viral MRN inhibitors might be enriched at adenotelomeres to facilitate initial MRN targeting, whether they might engage MRN through disordered peptide motifs analogous to those found at cellular telomeres, and whether they might also locally and directly suppress MRN activity.

Strikingly, given the unconventional nature of *D. melanogaster* telomeres, ATM activation by MRN—rather than its inhibition—is required for Drosophila telomere protection [105, 106, 107], possibly by promoting the correct recruitment of other telomere proteins through an unclear mechanism [108]. Since the fly telomere protein HOAP interacts with MRN subunits [109], it is conceivable that telomere proteins tightly control MRN functions at *Drosophila* chromosome ends, switching MRN toward a protective role, possibly through protein– contacts that remain to be elucidated.

## 5 Multiple Strategies for Shielding Eukaryotic Telomere DNA

Inactivation of the MRN‐inhibitory BAT/MIN and iDDR motifs leads to rather mild telomere protection defects in yeast and mammals [81, 82, 83], pointing to the existence of complementary protection pathways. Here we define telomere shielding as the physical coverage of telomeric DNA by proteins that sterically restrict access to DNA‐processing factors. Shielding operates on at least three levels: through the Myb‐domain telomere‐repeat binding protein layer, through end‐capping proteins at the telomere tip, and through higher‐order telomeric superstructures such as the T‐loop.

### 5.1 Basal Shielding by Myb‐Domain Proteins

Shielding by the basal telomeric protein layer is best characterized in budding yeast, where Rap1_Sc_ confers significant protection even in the absence of its telomeric partner proteins [110, 111, 112, 113]. Rap1 arrays effectively protect against NHEJ and resection both in vivo and in vitro, likely by preventing DNA end sensing/access by MRN and associated factors [88, 112]. This protection depends on several parameters, consistent with the architecture of native telomeres (Figure 4B): (i) array length, with maximum protection requiring arrays close to native telomere size; (ii) array density, as gaps between binding sites drastically weaken protection; (iii) proximity to the DNA end; and (iv) and subnanomolar Rap1 DNA‐binding affinities for telomeric sites [88]. Notably, Rap1 can be partially substituted by other high‐affinity DNA‐binding proteins such as LacI, suggesting that protection is primarily a function of tight, dense DNA occupancy [88]. Tandem Rap1 binding also stiffens the DNA, which may further impede DNA processing [114, 115]. Whether the basal protein layers of other organisms — such as TRF1/2 in mammals or Taz1 in *S. pombe*, which also bind telomeric DNA with high affinity [116] — provide analogous shielding remains an important open question. Even more enigmatic are *D. melanogaster* telomere proteins HipHop and HOAP [108, 117], which lack Myb domains yet can spread over 10 kb at chromosome ends, apparently independently of telomere sequence [108], and might play an analogous shielding role despite this fundamentally different mode of recruitment. The estimated average length of a reconstituted yeast Rap1 array (close to native telomere size) is about 90 nm [114], while mammalian telomeres, which adopt a three‐dimensional structure are estimated to exhibit an average diameter of about 180 nm (see below) [118]. These dimensions approach or exceed the estimated ∼60 nm length of eukaryotic RAD50 coiled coils [119]. Thus, in addition to physically shielding chromosome extremities (Figure 4B), telomeres are likely to challenge MRN's obstacle‐bypass abilities (Figure 2), preventing the complex from loading internally, far from the telomere extremity. Whether such shielding mechanisms also exist in bacteria is unclear (see below) [17].

### 5.2 Chromosome End‐Capping: KU Hijacking and Coating the Single‐Stranded Overhang

Two protein complexes cap the telomere extremity, KU (Ku70/Ku80) and CST (CTC1‐STN1‐TEN1 in humans; Cdc13‐Stn1‐Ten1 in budding yeast) (Figure 4C). KU is a ring‐shaped complex that encircles DNA ends and acts as a major NHEJ factor at double‐strand breaks, promoting repair by restricting 5' resection and recruiting core NHEJ factors including LIG4 and DNA‐PK in mammals [120, 121, 122, 123]. Strikingly, KU is also recruited to telomeres, where it prevents chromosome end hyper‐resection in yeast, plants, and mammals [82, 124, 125, 126, 127, 128, 129], while its NHEJ‐promoting functions must be locally suppressed. In budding yeast, Rap1 and KU bind together at the telomeric extremity, forming a ternary complex in which Rap1 acts as a doorstop, preventing the ∼25 bp inward translocation of KU required for NHEJ, thereby restricting access of both NHEJ and resection factors to telomeric DNA [130]. In mammals, RAP1, recruited by TRF2, contacts KU through its BRCT domain, preventing KU from interacting with LIG4 and thus inhibiting NHEJ [131], explaining the previously elusive role of human RAP1 at telomeres [132, 133, 134]. It remains to be determined whether mammalian RAP1‐TRF2 also restricts KU inward translocation, and whether additional protein motifs prevent KU from recruiting its NHEJ partners in yeast. Since KU homologs exist in bacteria [135], this complex may have been co‐opted early in evolution to contribute to telomere protection.

The single‐stranded telomeric overhang is further protected by CST, an RPA‐like complex that directly binds single‐stranded telomeric repeats and prevents their exonucleolytic processing [136, 137, 138, 139]. CST also recruits Polα‐primase for lagging strand re‐synthesis, counteracting resection following replication, or following telomerase activity [1, 136, 140, 141, 142, 143]. In mammals, POT1 (POT1A/POT1B in mice) additionally shields the ssDNA overhang against recognition by the ATR checkpoint kinase, through its interaction with TPP1 and possibly by sterically excluding RPA from telomeres [1, 144, 145].

### 5.3 Higher‐Order Telomeric Structures

Finally, telomeres in several species (including mammals, plants and nematodes) can form a structure called the T‐loop (Telomeric loop) [146, 147], which further restricts access of DNA repair factors to chromosome ends, effectively protecting telomeres from inappropriate DNA damage signaling, NHEJ and likely exonuclease action [1, 89, 90]. The T‐loop (detected in about 20%–40% of telomeres [146, 147, 148]) is formed by the invasion of the terminal 3' single‐stranded overhang into the upstream double‐stranded telomeric duplex, creating a large lariat structure possibly harboring a displacement loop (D‐loop) at the junction [147] (Figure 4D). T‐loop formation requires TRF2 [146, 147, 148, 149], possibly via its tetramerization, as artificial tetramerization of TRF1 confers telomere protection [150]. The T‐loop must however be disassembled during replication to facilitate fork progression, a process that would be regulated by de‐phosphorylation and subsequent re‐phosphorylation of TRF2 on serine 365 [151]. During this process, other telomere proteins are likely essential to maintain end protection.

The T‐loop structure, by burying the 3' overhang extremity into the double‐stranded telomeric track should in principle prevent telomeric DNA end sensing and recognition by MRN, effectively inhibiting all downstream activities of the complex. Whether a wide opening of RAD50 coiled‐coil arms could allow MRN to bypass the whole T‐loop and allow internal loading of the complex in certain conditions, for example, if the T‐loop is small, remains to be assessed. Similarly, the T‐loop should prevent NHEJ factors such as KU and LIG4 from productive loading and from forming an NHEJ synapsis. Moreover, the T‐loop protective effect would synergize with other telomere shielding mechanisms described above such as protein–protein interactions (e.g., TRF2 iDDR) or basal shielding by Myb‐proteins (e.g., TRF2/TRF1), together resulting in robust telomere protection. For more information regarding the T‐loop and telomere protection in mammals, we refer the reader to specialized reviews [1, 89, 90].

## 6 A Single Protein Ensures Replication and Protection of a Bacterial Telomere

The HP and TIR classes of prokaryotic telomeres address the end replication problem through their intrinsic architecture, as described above. They also hinder access of cellular exonucleases such as RecBCD (and DNA ligases), by forming a DNA hairpin and by covalently connecting the 5’ end of DNA to a protein, respectively (Figure 4C). However, how bacterial telomeres protect themselves from the MR_bact_ nuclease, which can readily process DNA ends with protein blocks and hairpins [70, 71, 152] (Figure 2) had remained elusive. Recent work on HP telomeres of the *E. coli* bacteriophage N15 has shed light on this question, revealing that the phage telomere resolvase TelN itself, responsible for generating hairpin telomeres in the first place [44, 52], is also a direct inhibitor of the MR_bact_ complex [17]. It is thus a dual‐function telomere protein. Linearization of the chromosome using N15 HP telomeres is well tolerated in *E. coli* [72], however, it was recently found to be lethal in *B. subtilis*, unless MR_bact_ was genetically inactivated [17]. A similar observation has been made when substituting native telomeres for HP telomeres in yeast, which are tolerated well, but only when MRX is mutated [153]. The HP telomeres also become unstable in *E. coli* when TelN protein is absent, unless MR_bact_ is also absent, altogether demonstrating that TelN protects phage‐derived DNA ends from *E. coli* MR‐mediated degradation but not from *B. subtilis* MR_bact_ or yeast MRX [17, 153, 154]. Importantly, this protective function is independent of TelN's telomere resolvase activity, identifying it as a distinct and separable function of the protein. Furthermore, protection does not require the carboxy‐terminal domain of TelN, which is the least conserved region of the protein with no known function, suggesting that, at least, a minimal protection mechanism resides within its highly conserved region.

TelN‐mediated protection is dose‐dependent in vitro [17]. Non‐telomeric DNA can also be partially protected against MR_bact_ in vitro, while protection requires TelN binding to its telomeric sequence in vivo. For instance, TelN fails to protect the HP telomeres derived from the related *Yersinia enterocolitica* phage PY54, indicating that sequence‐specific targeting to the correct telomeric substrate is required for effective protection in a cellular context. However, TelPY (from PY54 phage) can protect its own hairpin telomeres from *E. coli* MR_bact_, despite significant sequence divergence from TelN, possibly through steric hindrance rather than specific protein–protein contacts. Moreover, TelN is unable to protect its cognate telomere against the yeast MRX complex in vitro, implying that it fails to sterically block the larger yeast nuclease or that species‐specific interactions with the bacterial MR complex are required for efficient inhibition in vitro. TelN binds DNA with only moderate affinity (estimated *K*d ∼200–300 nM), independently of DNA structure or length. Together, these observations suggest that telomere resolvases have evolved at least two distinct modes of MR inhibition: specific protein–protein interactions and sequence‐independent steric shielding.

How the relatively small TelN protein may shield the HP telomere from the large MR_bact_ nuclease remains unclear. The estimated TelN diameter is ∼11 nm (based on an AlphaFold3 prediction [17]), which is smaller than the KU complex (diameter: ∼12 nm [122]) that can readily be bypassed by MRN [70, 71]. Thus, even considering that prokaryotic RAD50 proteins possess shorter coiled‐coil arms than eukaryotic counterparts (∼25 nm instead of ∼60 [119, 155]), MR_bact_ would be expected to be able to bypass a TelN monomer bound to the HP telomere and load close to the phage hairpin telomere end. Future research should thus investigate how MR_bact_ is controlled or inactivated by telomere resolvases. Exploring whether prokaryotic telomeric proteins possess MR‐inhibitory peptide motifs analogous to those in eukaryotes (Figure 4A) will be of great interest. They might also (1) nucleate/spread on DNA ends to form an array, shielding the DNA akin to the basal protein layer of eukaryotic telomeres (Figure 4B), and (2) promote higher‐order nucleoprotein structures that might contribute an additional layer of end protection (Figure 4D). Structural studies characterizing the architecture of hairpin telomere–resolvase complexes, and identifying direct contact points with MR_bact_, will thus be essential to fully elucidate the mechanism of MR inhibition in bacteria. Little is known about whether and how TIR telomeres protect themselves from MR.

## 7 A Role of Biological Conflicts in Shaping Telomere Evolution

In most bacteria chromosomes are circular, making the DNA ends of mobile genetic elements attractive targets for bacterial defense systems. Indeed, multiple systems use free DNA ends as signals to detect and restrict foreign DNA. DNA repair proteins such as MR, RecBCD, and AddAB degrade linear DNA entering the cell, while dedicated defense systems such as Shedu, Gabija, and OLD nucleases similarly exploit free DNA ends or the absence of RecBCD activity as indicators of nonself DNA [156, 157]. Lamassu, a defense system closely related to MR, likely uses its Rad50‐like subunit to sense DNA‐end and hairpin signatures associated with mobile genetic elements, ultimately triggering cell death to protect neighboring cells from infection [158, 159, 160, 161], possibly through oligomerization of highly variable effectors. Counter‐defense strategies have evolved in response: the phage lambda Gam protein, for instance, inhibits RecBCD by mimicking DNA [162]. In eukaryotes, free DNA ends are equally potent danger signals, with core repair factors such as MRN and KU [163, 164, 165] contributing together with specialized immune factors to the sensing of DNA damage and cellular infection [166, 167, 168]. It is therefore not surprising that telomeres have evolved elaborate mechanisms to prevent self‐recognition by these same factors (Figures 3, 4), while permitting just enough MRN activity to signal critically short telomeres and trigger senescence before protective functions are lost.

The presence of linear chromosomes creates an additional challenge: preventing mobile genetic elements from exploiting telomere signatures to evade innate immune surveillance. Since DNA repair complexes such as MRN, whose activity is normally suppressed at telomeres, also provide innate immunity against invasive genetic elements [3], an MGE bearing telomere‐like terminal structures could thus conceivably mimic a “self” chromosome end to escape MRN‐mediated detection. In bacteria, simple telomeres are compatible with MGE life cycles and are indeed exploited by bacteriophages and phage‐plasmids [21, 53], potentially explaining why linear chromosomes remain rare among bacteria, while viruses encode many factors that subvert DNA sensors [3, 169]. Yet the evolutionary arms race between cells and invasive genetic elements is also a source of molecular innovation [170, 171]. Strikingly, DRT10, a defense‐oriented reverse transcriptase that produces repetitive cDNA from an RNA template to counteract phage infection, shares evolutionary links with the human telomerase catalytic subunit TERT, and can even produce telomere repeats in vivo when provided with a telomeric RNA template [172, 173]. This phylogenetic link raises the intriguing possibility that telomerase originated from a bacterial defense reverse transcriptase.

## 8 Conclusions

Telomeres support chromosome end replication and solve the challenge of end protection, which is universal to any organism harboring linear DNA, including viral entities. The solutions that have emerged across the tree of life are remarkably diverse and include the coating of DNA ends with shielding proteins and directly inhibiting enzymes that pose the greatest threats to telomere integrity.

A central insight is the extent to which convergent evolution has shaped telomere protection. Unrelated telomere proteins in budding yeast, fission yeast, and mammals have independently evolved short peptide motifs that dock onto the same surface of the Rad50 ATPase head to suppress MR activity, driven by the universal imperative to restrain a nuclease capable of partially overcoming DNA shielding proteins. Bacteriophages have likewise evolved telomere proteins that counteract MR activity to permit phage propagation. MR inhibition may therefore represent an ancestral function of telomeres, and possibly a prerequisite for the stable existence of any linear chromosome.

The evolutionary arms race may also reframe the origins of telomere components themselves. The likely descent of telomerase from a bacterial defense reverse transcriptase, and the deep connections between DNA end‐sensing defense systems and telomere regulation, suggest that telomere biology was shaped, and continues to be shaped, by the ongoing conflict between host genomes and the mobile genetic elements that exploit, evade, and coevolve with them. Understanding telomere evolution through this lens may thus illuminate not only the origins of chromosome end protection, but also the broader principles of how genomes maintain integrity in the face of constant molecular conflict.

## Author Contributions

F.R.‐H. and S.G. conceptualized the manuscript. F.R.‐H. wrote the first draft; F.R.‐H. and S.G. revised the manuscript.

## Funding

This work was supported by an SNSF project grant (320030‐227915 to S.G.). F.R.‐H. was supported by an EMBO Postdoctoral fellowship (ALTF 302–2022).

## Conflicts of Interest

The authors declare no conflicts of interest.

## Data Availability

Data sharing is not applicable to this article as no new data was generated.
